# Determinants of Utilization of Antenatal Care Services Among Women of Childbearing Age in Jigawa State, Nigeria

**DOI:** 10.3389/ijph.2024.1607385

**Published:** 2024-09-17

**Authors:** Abdulwali Sabo, Majdi M. Alzoubi, Abdulhamid Yaro Saidu, Usman Sunusi Usman, Ibrahim Musa Saulawa, Khalid Al-Mugheed, Sally Mohammed Farghaly Abdelaliem, Amany Anwar Saeed Alabdullah

**Affiliations:** ^1^ Department of Public and Environmental Health, Faculty of Basic Medical Sciences, College of Medicine and Allied Medical Sciences, Federal University Dutse, Dutse, Nigeria; ^2^ Faculty of Nursing, Al-Zaytoonah University of Jordan, Amman, Jordan; ^3^ Department of Community Medicine, Faculty of Clinical Sciences, College of Medicine and Allied Medical Sciences, Federal University Dutse, Dutse, Nigeria; ^4^ Faculty of Nursing, Riyadh Elm University, Riyadh, Saudi Arabia; ^5^ Department of Nursing Management and Education, College of Nursing, Princess Nourah bint Abdulrahman University, Riyadh, Saudi Arabia; ^6^ Department of Maternity and Paediatric Nursing, Riyadh, Saudi Arabia

**Keywords:** antenatal care utilization, determinant, knowledge, attitude, practice

## Abstract

**Introduction:**

Antenatal care (ANC) services play a crucial role in safeguarding the health of pregnant women during their reproductive years. This study aimed to evaluate the primary factors influencing the utilization of ANC among women of childbearing age in Isari town, Jigawa State.

**Methods:**

We conducted a cross-sectional study among 400 mothers of childbearing age, selecting them using a simple random sampling method. Data were collected using interviewer-administered questionnaires. The statistical analyses performed were descriptive analysis, Pearson’s chi-square test, and binary logistic regression analysis.

**Results:**

The majority of respondents (92.5%) indicated awareness of ANC, with a significant proportion expressing the necessity of ANC services (85.7%). 57.8% of the respondents indicated attending ANC services at least four times during pregnancy. Furthermore, the number of visits has a significant relationship with age (*P* < 0.001), educational level (*P* = 0.003), occupation (*P* = 0.043), mother’s knowledge of pregnancy danger signs (*P* = 0.001), and husband’s support for ANC (*P* < 0.001).

**Conclusion:**

Enhancing ANC utilization will necessitate focusing on women residing in rural areas and those with limited educational attainment.

## Introduction

For numerous years, the global community has shown a profound interest in advancing women’s optimal health and reducing rates of maternal and child mortality [1, 2]. Maternal and child healthcare received utmost priority in both the Millennium Development Goals (MDGs) and, more recently, the Sustainable Development Goals (SDGs), underscoring this targeted focus [3]. Between 1990 and 2015, the MDGs initiative led to substantial improvements in maternal and child healthcare worldwide, as evidenced by reductions of 43.9% and 48% in the maternal mortality ratio (MMR) and under-five mortality rate (U5MR), respectively [1, 2]. Despite these notable strides, significant disparities persist within and among various demographic groups regarding maternal, neonatal, and childhood mortality rates in numerous developing nations [2, 3].

Nigeria remains a major contributor to global under-five, neonatal, and maternal mortality rates, representing approximately 13%, 9.4%, and 14% of all deaths, respectively [4, 5]. These poor indices could be attributed to the poor utilization of maternity healthcare services [6, 7]. Recent studies have indicated a relationship between the usage of maternal healthcare services, the maternal mortality ratio (MMR), and infant mortality rates [8, 9]. Studies have specifically highlighted the protective effect of antenatal care (ANC) attendance against newborn mortality, with nations exhibiting poor ANC attendance experiencing higher MMRs [9, 10]. ANC, a cornerstone of the “safe motherhood initiative,” continues to represent a significant public health intervention aimed at averting maternal and newborn deaths globally, as indicated by the mounting body of evidence on the subject [11].

ANC, sometimes referred to as prenatal healthcare, involves assessing both the mother and foetus throughout pregnancy to optimize their health outcomes [12]. The World Health Organization (WHO) promotes a “focused” ANC package, advocating for a minimum of four prenatal visits. This includes counseling, supplementation (iron and folic acid), medical screenings, vaccinations, and malaria preventive treatment, aiming for safe pregnancy outcomes [12, 13]. However, WHO now recommends at least eight ANC visits as opposed to four visits to promptly identify issues and significantly reduce perinatal and maternal mortality rates [14].

Despite the availability and importance of prenatal care, 36% of pregnant women worldwide attend antenatal clinics fewer than four times during their pregnancy, while some do not attend at all [12]. In sub-Saharan Africa, just over 44% of pregnant women visit ANC clinics four or more times, compared to 71% who attend at least once [12]. The widespread underutilization of prenatal care services may contribute to the persistently high rates of maternal and perinatal deaths in Nigeria. Although approximately 61% of pregnant women attended ANC, this marks only a modest improvement of 3% from the Nigeria Demographic Health Survey (NDHS) 2008 [7, 12].

Factors influencing the utilization of ANC in Nigeria include maternal age, working status, education level of both the mother and her husband, wealth index, rural-urban residence, region of residence, and religion [4, 7]. Nigeria exhibits significant sociocultural and socioeconomic gaps between rural and urban areas, with rural and economically disadvantaged communities experiencing higher rates of underutilization of ANC services [7]. For this reason, Jigawa State, being one of the poorest states in Nigeria, had previously launched the Jigawa State Free Maternal Child Health Program (JSFMCHP) through the Ministry of Health to address the state’s alarming rates of maternal and infant mortality. This program aims to enhance access to and utilization of maternal and child health services, particularly targeting areas facing financial constraints [15].

Certainly, optimal utilization of maternal healthcare services would lead to a reduction in maternal death rates and pregnancy-related complications [14]. The relatively low maternal mortality ratios observed in high-income nations, as opposed to low-income countries like Nigeria, have been partially attributed to the use of modern obstetric services [14]. The utilization of health services is a complex behavioral phenomenon, and studies have demonstrated that the use of health services is influenced by social structure, health beliefs, individual characteristics of users, and the availability, quality, and cost of services [16–18]. Consequently, this study seeks to identify the level of ANC utilization and its associated factors among married women of childbearing age in Jigawa State. We expect that the results will provide valuable insights for designing targeted interventions to improve ANC attendance, thereby assisting Nigeria in achieving Sustainable Development Goal 3.

## Methods

### Study Area

Isari town is a rural community located in the Dutse local government area of Jigawa State, situated in the northern part of Nigeria. Its geographical coordinates are 12°1′30″North and 9°18′31 East. Isari town has only one primary healthcare center, where approximately 10 healthcare providers offer ANC services. Although there were no documented characteristics of Isari town, Jigawa State is classified among those with a relatively high severity and incidence of poverty in the country. It has a gross *per capita* income of N35,000 per annum (US$290), which is below the national average. Currently, Jigawa State is among the poorest states in Nigeria, with a total fertility rate of about 6.2 children per woman of childbearing age, slightly above the national average. Additionally, 90% of the state’s population resides in rural areas. The gender distribution is almost equal, with males making up 50.8% and females making up 49.2% of the population. This pattern of population distribution is consistent across various constituencies and between urban and rural areas.

### Sample Size Estimation

The sample size was calculated using the single proportion formula [19]. Based on an estimated proportion (P) of 0.307, reflecting the anticipated rate of ANC usage in northwest Nigeria [7], a margin of error (E) of 0.05, and a Z-value of 1.96 corresponding to a 95% confidence interval, the initial estimate yielded 327 samples. To accommodate potential missing values and errors in data entry, the adjusted sample size was recalculated to be 408, incorporating a 20% dropout rate.

### Research Instrument

This study employed a semi-structured questionnaire divided into four sections, based on previous research [20]. The first section covered sociodemographic factors; the second section addressed mothers’ knowledge; the third section explored mothers’ attitudes regarding ANC services; and the fourth section examined mothers’ practices related to ANC utilization. However, to ensure its validity for the targeted population, we assessed content validity by consulting six experts: two lecturers from community medicine, two from public health, and two from questionnaire development. They evaluated the relevance of each question to its respective factor, ensuring the questionnaire effectively captured the intended information. To assess content validity, we calculated both the item content validity index (I-CVI) and the scale content validity index (S-CVI). An I-CVI and S-CVI of 0.83 or higher were considered indicative of satisfactory content validity [21]. The S-CVI values were 1.00 for the knowledge factor, 1.00 for the practice factor, and 0.97 for the attitude factor. Additionally, the I-CVI for all questions ranged from 0.83 to 1.00. These CVI values exceeded the standard cut-off, confirming the questionnaire’s content validity [21].

In this study, a total of 76 medical students (59.2% males and 40.8% females) aged 20–25 years were employed for the data collection process. The students underwent 3 days of training on how to translate the questions into Hausa prior to data collection. Subsequently, lecturers from the department of community medicine, who are both public health experts and fluent in Hausa and English, assessed the students’ translation quality and found that the students’ translation quality was acceptable. All the students were fluent in both English and Hausa. Consequently, because the majority of the study participants do not speak English, interviews were conducted in Hausa.

### Inclusion Criteria and Exclusion Criteria

The study included married mothers within the childbearing age range (15–49 years). In Nigeria, the reproductive age is commonly defined as between 15 and 49 years [22, 23]. Demographic and health studies frequently use this age range to encompass the years during which women are most likely to conceive and give birth [22]. Those who refused to participate, were seriously ill, or fell outside the childbearing age range were excluded. Furthermore, we included only married mothers of childbearing age due to the predominant Islamic and Hausa culture in the state, which assumes that only married women are expected to have pregnancies. This cultural context makes them the primary beneficiaries of ANC services.

### Data Collection

A household census was carried out in Isari town, identifying a total of 1,300 households. From this pool, 500 households were randomly selected using a simple random sampling method. Subsequently, researchers visited the chosen residences to conduct interviews with eligible and consenting mothers of reproductive age living in these households. If the respondents failed to meet the study’s inclusion criteria, the team proceeded to the next household until they achieved the desired sample size. Data collection occurred between 3 January and 10 February 2022. To ensure the accuracy of the data, researchers conducted daily double-checks on the responses. Any issues that arose were promptly addressed by the research team.

### Study Design and Location of Study

The study was a cross-sectional study conducted in Isari town, situated in Jigawa State, Northwest Nigeria, as part of the Community-Based Medical Education Field Posting (CBME and SP) program for third-year medical students at the Federal University of Dutse, Jigawa State. Additionally, prior to the data collection, the lecturers from the department of community medicine, accompanied by all the students, made a courtesy visit to the village to introduce themselves and build rapport with community stakeholders. This visit aimed to ensure a smooth survey process, raise awareness, garner community support, and collect high-quality data that accurately reflects the perspectives and experiences of the community members.

### Statistical Analysis

Initially, we conducted data cleaning to rectify missing values and erroneous data entries. The statistical analyses applied in this study comprised descriptive analysis, the Pearson Chi-square test, and binary logistic regression analysis, utilizing Statistical Product and Service Solution (SPSS) version 27. We used descriptive analysis to present frequencies and percentages. The Pearson Chi-square test was performed to determine the association between the participants general characteristics (age group, education, occupation, knowledge about the dangers of pregnancy, and whether your husband supports your ANC visit) and the number of ANC visits (i.e., <4 vs. ≥ 4). We conducted a binary logistic regression analysis to identify significant predictors (age group, education, occupation, knowledge about the dangers of pregnancy, and whether your husband supports your ANC visit) associated with any ANC visits (yes vs. no). In the initial stage of logistic regression, simple logistic regression was performed to determine the crude odds ratio of predictors. Factors with *p*-values less than 0.25 were deemed important and included in the multiple logistic regression analysis to remove any confounders and obtain an adjusted odds ratio. Bursac, Gauss [24] stated that using a cutoff point of 0.05 might fail to identify important variables. Therefore, variables with a *p*-value <0.25 should be included in the multiple logistic regression analysis [24]. Both forward LR and backward LR methods were employed in the multiple logistic regression, and the final model was run using the enter method.

Logistic regression is commonly used to predict a binary outcome (e.g., success or failure, yes or no) [25]. The primary focus is on the accuracy and reliability of these predictions rather than on how sensitive the model is to changes in specific variables [25]. Instead of sensitivity tests, logistic regression analysis focuses on diagnostic checks for model fit. These include looking at how variables interact with each other, running Hosmer and Lemeshow tests, checking the accuracy of classification, and checking the area under the receiver operating characteristic curve (AUC) [25, 26]. Therefore, we assessed the adequacy of the study’s final model using these parameters. All five predictors (age group, education, occupation, knowledge about the dangers of pregnancy, and whether your husband supports your ANC visit) were retained in the final model.

## Results

### General Characteristics of the Study Respondents

Table 1 presents the general characteristics of the study respondents. A total of 400 married women participated in the study. The majority of the respondents were between the ages of 15 and 34 (82%) and Hausa (99.5%). About half of the participants possessed a non-formal education (47.5%) and were unemployed (41.7%). Furthermore, 100% of the participants identified as Muslims.

**TABLE 1:** General characteristics of the study participants (n = 400). Jigawa, Nigeria (2024).

Variable	Category	N	%
Age group	15–25 25–34 35–44 >44	166 162 56 16	41.5 40.5 14.0 4.0
Ethnicity	Hausa Fulani	398 2	99.5 0.5
Education	Primary Secondary Tertiary Non-formal None	84 62 33 190 31	21.0 15.5 8.3 47.5 7.7
Occupation	Civil servant Business Others None/Housewives	68 154 11 167	17.0 38.5 2.8 41.7
Religion	Islam	400	100

N, number.

Table 2 presents mothers’ knowledge about the ANC. The majority of respondents (92.5%) indicated awareness of ANC, with a significant proportion acknowledging it as a service for pregnant women (85.7%). More than half of the respondents advocated for mothers to attend ANC services at least four times during pregnancy (67.7%) and identified nutrition and diet (74.3%) and birth preparedness and breastfeeding (57.0%) as services provided during ANC. A large proportion of respondents recognized folic acid as the medication administered during ANC (80.0%), followed by anti-malaria drugs (14.2%). A significant proportion of respondents demonstrated knowledge of pregnancy danger signs (83.0%), with headaches (58.5%) and high fevers (60.8%) being frequently reported. Additionally, approximately half of the respondents highlighted prevention of pregnancy complications (59.0%) and improvement in child and maternal health (56.0%) as the benefits of ANC. The majority suggested that ANC should ideally commence at least 6 months into pregnancy (83.2%).

**TABLE 2:** Mothers knowledge about antenatal care. Jigawa, Nigeria (2024).

Question	Category	N	%
Do you know ANC services?	No Yes	30 370	7.5 92.5
If Yes, what is ANC?	Service giving to a couple Service-giving to pregnant women Service-giving to women of childbearing age	21 317 32	5.7 85.7 8.6
Where is the services given?	Health institution Traditional places Home Don’t know	369 10 1 20	92.2 2.5 0.3 5.0
How many times should mothers attend ANC services during pregnancy?	<4 ≥4 Don’t know	109 271 20	27.3 67.7 5.0
Service rendered during ANC include (multiple response apply)	Nutrition and Diet Birth plan Birth preparedness and breastfeeding HIV testing TT Toxoid	297 124 228 116 132	74.3 31.0 57.0 29.0 33.0
What are the drugs given during ANC?	Folic acid Anti-malaria Ferrous sulphate Anti-retroviral drugs Don’t know	320 57 2 1 20	80.0 14.2 0.5 0.3 5.0
Do you know about danger sign of pregnancy?	No Yes	68 332	17.0 83.0
If Yes, What are the danger signs of pregnancy? (Multiple response apply)	Headache Vaginal bleeding High fever Decrease in foetal movement None of the above	234 125 243 115 22	58.5 31.3 60.8 28.7 28.7
Importance of ANC (Multiple responses apply)	Prevent complication in Pregnancy Prevent maternal and child mortality Improve the health of the mother and child Increase income Others	236 126 224 41 9	59.0 31.5 56.0 10.3 2.3
What is the ideal time to start ANC? (Weeks)	<6 months ≥6 months Don’t know	47 333 20	11.8 83.2 5.0

N = number.

Table 3 presents mothers’ attitudes toward the ANC. The majority of respondents (85.7%) expressed the necessity of ANC services, with a significant proportion acknowledging the benefits of vitamin supplements and folic acid tablets for the foetus (83.0%). A large proportion of respondents indicated their willingness to have their blood pressure checked by health workers (92.8%) and recognized the importance of early ANC booking for pregnant women (90.7%). Furthermore, the majority of respondents reported receiving support for ANC from their husbands (93.7%), with 85.9% receiving reminders from them. However, only 35.7% stated that their husbands accompanied them to ANC appointments.

**TABLE 3:** Mothers attitude toward antenatal care services. Jigawa, Nigeria (2024).

Question	Category	N	%
Are ANC services necessary?	No Yes Don’t know	37 343 20	9.3 85.7 5.0
Do you believe that vitamin supplement and folic acid tablet is good for the foetus?	No Yes Don’t know	48 332 20	12.0 83.0 5.0
Would you allow the health workers to check your BP?	No Yes	29 363	7.2 92.8
Is early antenatal booking well for pregnant women?	No Yes	37 363	9.3 90.7
Does your husband support your ANC visit?	No Yes	25 375	6.3 93.7
If yes, has your husband ever remind you of your ANC visit?	No Yes	53 322	14.1 85.9
If yes, has your husband ever accompanied your ANC visit?	No Yes	241 134	64.3 35.7

N, number.

Table 4 presents mothers’ practices toward the ANC. Over half of the respondents (60.2%) stated that they attended ANC during their most recent pregnancy, with 67.2% reporting that they initiated ANC services within the first 10 weeks of pregnancy. Additionally, 57.8% of the respondents indicated attending ANC services at least four times during pregnancy, and all respondents (100%) mentioned health facilities as the location where they received ANC services.

**TABLE 4:** Mothers practice toward antenatal care services. Jigawa, Nigeria (2024).

Question	N	%
Have you attended antenatal care during the last pregnancy? No Yes	159 241	39.8 60.2
If yes, at what age (weeks) of pregnancy did you attend ANC? ≤10 >10	79 162	32.8 67.2
How many times did you attend ANC during pregnancy? <4 ≥4	231 169	57.8 42.2
Where do you receive the service? Health facility	241	100

N, number.

Table 5 presents the relationship between maternal characteristics and the number of ANC visits. The results show that the number of visits has a significant relationship with age (*P* < 0.001), educational level (*P* = 0.003), occupation (*P* = 0.043), mothers knowledge of pregnancy danger signs (*P* = 0.001), and husbands support for ANC (*P* < 0.001). Specifically, individuals aged 25–34 years (59.3%), those with tertiary education (57.6%), civil servants (55.95%), individuals knowledgeable about pregnancy danger signs (46.1%), and those receiving ANC support from their husbands (45.1%) exhibited the highest rates of ANC attendance.

**TABLE 5:** Relationship between maternal characteristics and the number of antenatal care visits. Jigawa, Nigeria (2024).

Characteristic	Category	<4 visits N (%)	≥4 visits N (%)	P
Age group				<0.001
	15–25	126 (75.9)	40 (24.1)	
	25–34	66 (40.7)	96 (59.3)	
	35–44	26 (46.4)	30 (53.6)	
	>44	13 (81.3)	3 (18.2)	
Education				0.003
	Primary	45 (53.6)	39 (46.4)	
	Secondary	32 (51.6)	30 (48.4)	
	Tertiary	14 (42.4)	19 (57.6)	
	Non-formal	113 (59.5)	77 (40.5)	
	None	27 (87.1)	4 (12.9)	
Occupation				0.043
	Civil servant	30 (44.1)	38 (55.9)	
	Business	87 (56.5)	67 (43.5)	
	Others	7 (63.6)	4 (36.4)	
	None/Housewives	107 (64.1)	60 (35.9)	
Do you know about danger sign of pregnancy?				0.001
	No	52 (76.5)	16 (23.5)	
	Yes	179 (53.9)	153 (46.1)	
Does your husband support your ANC visit?				<0.001
	No	25 (100)	0 (0)	
	Yes	206 (54.9)	169 (45.1)	

N, number.

Table 6 presents the factors associated with any ANC visits using simple and multiple logistic regression analyses. We used the first practice question, “Have you attended antenatal care during the last pregnancy?” to categorize those who had attended any ANC visits and those who had not. Among the 400 study participants, 159 (39.8%) responded no, and 241 (60.2%) responded yes. For age, those 25–34 years old were 8.4 times more likely to visit ANC compared to those 15–24 years old [OR = 8.38 (95% CI: 4.77, 14.70), *P* < 0.001]; those 35–44 years old were 11.9 times more likely to visit ANC compared to those 15–24 years old [OR = 11.93 (95% CI: 5.13, 27.74), *P* < 0.001]; and those above 44 years old were 4.1 times more likely to visit ANC compared to those 15–24 years old [OR = 4.13 (95% CI: 1.30, 13.13), *P* = 0.016]. For education, those with secondary education were 36% less likely to visit ANC compared to those with primary education [OR = 0.64 (95% CI: 0.27, 1.51), *P* = 0.305]; those with tertiary education were 53% more likely to visit ANC compared to those with primary education [OR = 1.53 (95% CI: 0.46, 5.11), *P* = 0.488]; those with non-formal education were 27% less likely to visit ANC compared to those with primary education [OR = 0.73 (95% CI: 0.38, 1.43), *P* = 0.360]; and those with none were 79% less likely to visit ANC compared to those with primary education [OR = 0.21 (95% CI: 0.07, 0.65), *P* = 0.007]. For occupation, the civil servants were 4.2 times more likely to visit ANC compared with none or housewives [OR = 4.19 (95% CI: 1.73, 10.18), *P* = 0.002]; those doing business were 19% more likely to visit ANC compared with none or housewives [OR = 1.19 (95% CI: 0.69, 2.07), *P* = 0.536]; and others were 2.1 times more likely to visit ANC compared with none or housewives [OR = 2.13 (95% CI: 0.41, 11.13), *P* = 0.371]. Participants with knowledge about pregnancy danger signs were 80% more likely to visit ANC compared to those without [OR = 1.80 (95% CI: 0.93, 3.48), *P* = 0.083]. Those receiving ANC support from their husbands were 4.8 times more likely to visit ANC compared to those without support [OR = 4.84 (95% CI: 1.37, 17.05), *P* = 0.014].

**TABLE 6:** Factors associated with any antenatal care visits (yes vs. no). Jigawa, Nigeria (2024).

Factor	Category	Any ANC visits		OR (95% CI)	*P*
		No N (%)	Yes N (%)		
Age group					
	15–24	113 (68.1)	53 (31.9)	1	
	25–34	29 (17.9)	133 (82.1)	8.38 (4.77, 14.70)	<0.001
	35–44	10 (17.9)	46 (82.1)	11.93 (5.13, 27.74)	<0.001
	>44	7 (43.8)	9 (56.3)	4.13 (1.30, 13.13)	0.016
Education					
	Primary	31 (36.9)	53 (63.1)	1	
	Secondary	26 (41.9)	36 (58.1)	0.64 (0.27, 1.51)	0.305
	Tertiary	6 (18.2)	27 (81.2)	1.53 (0.46.5.11)	0.488
	Non-formal	73 (38.4)	117 (61.6)	0.73 (0.38, 1.43)	0.360
	None	23 (74.2)	8 (25.8)	0.21 (0.07, 0.65)	0.007
Occupation					
	Civil servant	9 (13.2)	59 (86.8)	4.19 (1.73, 10.18)	0.002
	Business	64 (41.6)	90 (58.4)	1.19 (0.69, 2.07)	0.536
	Others	4 (36.4)	7 (63.6)	2.13 (0.41, 11.13)	0.371
	None/Housewives	82 (49.1)	85 (50.9)	1	
Do you know about danger sign of pregnancy?					
	No	42 (61.8)	26 (38.2)	1	
	Yes	117 (35.2)	215 (64.8)	1.80 (0.93, 3.48)	0.083
Does your husband support your ANC visit?					
	No	20 (80.0)	5 (20.0)	1	
	Yes	139 (37.1)	236 (62.9)	4.84 (1.37, 17.05)	0.014

N, number, OR, odds ratio; CI, confidence interval.

Furthermore, there was no significant interaction between all the predictors in the final model (*P* > 0.05), indicating no significant correlation between all the predictors. The Hosmer and Lemeshow Test (*P* = 0.110), classification accuracy = 75.8%, and AUC = 83.2%. This indicates that the final model has an adequate fit.

## Discussion

According to many studies, ANC plays a crucial role in mitigating the risks of high maternal mortality in developing nations by facilitating early diagnosis and treatment of illnesses and ensuring safe childbirth [3, 7, 15, 27]. Efforts to enhance ANC services have been ongoing for over three decades in lower income countries [27, 28]. These efforts include implementing user fee policies, providing education to women, promoting women’s empowerment through employment opportunities, and advocating for family planning [29]. Despite making substantial progress towards achieving MDGs 4 and 5, Nigeria remains a significant contributor to global maternal and child mortality rates [17, 30]. One contributing factor to this challenge is the limited access to maternal and child health services, primarily due to poverty and regional disparities [7, 17]. Hence, conducting research to explore the determinants of ANC utilization will provide essential evidence for shaping policies and planning programs aimed at improving maternal and child health outcomes.

The majority of participants (92.5%) demonstrated awareness of ANC, with a notable proportion (85.7%) possessing accurate knowledge about ANC services. This may be attributed to sustained government and agency campaigns aimed at promoting awareness of ANC services nationwide. The implications of ANC knowledge levels in Nigeria are profound for maternal and child healthcare outcomes. Having sufficient ANC knowledge is imperative for women to make well-informed decisions regarding their health and that of their unborn child [4, 7, 12]. Women who possess knowledge about ANC are more inclined to initiate ANC services early in pregnancy, adhere to recommended ANC visits, and adopt healthy behaviors throughout pregnancy, including proper nutrition and birth preparedness [12, 31, 32]. It is essential to recognize that ANC knowledge varies among different demographic groups and regions in Nigeria [3, 7, 12]. While some women exhibit a comprehensive understanding of ANC, others may lack awareness or hold misconceptions regarding its significance and services. Various factors, such as educational attainment, socioeconomic status, urban-rural disparities, and access to healthcare facilities, exert considerable influence on ANC knowledge among Nigerian women [3, 16, 27].

The study participants exhibit a favorable attitude toward ANC services, with a significant majority of respondents affirming the importance of ANC services. Additionally, a notable proportion acknowledges the benefits of vitamin supplements and folic acid tablets for foetal health. However, just over half of the respondents attended ANC during their most recent pregnancy, and indicated attending ANC services at least four times during pregnancy. This is below the desired threshold of 90% [33]. Contrasting with other observations in Nigeria, ANC utilization was documented at 59.0% in Kano [27], 54% according to the 2013 Nigeria Demographic and Health Survey [4], and 69.3% for the northwest region [7]. Other sub-Saharan African countries have reported relatively higher ANC coverage, including Ghana (78.2%), Benin Republic (58.2%), Liberia (78.1%), Sierra Leon (76%), Lesotho (70.4%), and Zimbabwe (64.8%) [4].

In the present study, individuals aged 25–34 years, those with tertiary education, civil servants, individuals knowledgeable about pregnancy danger signs, and those receiving ANC support from their husbands had the highest rate of at least four ANC visits. This aligns with prior research conducted in Nigeria, which identified factors such as the mother’s age, residential location (urban or rural), educational attainment of both the mother and her husband, the woman’s employment status, household wealth status, and enrolment in health insurance as influencing ANC utilization [4, 7, 27]. These factors are in line with those identified in a systematic review of the literature on the barriers to ANC utilization in developing nations [34].

The findings of the present study indicated that the odds of ANC utilization increase with age, among those with tertiary education. Similarly, previous studies in Nigeria have found that older age and higher levels of education for both mothers and their husbands increase the likelihood of ANC utilization [3, 4, 12, 27]. Comparable trends have been observed in other African countries, where education significantly influences healthcare service utilization [16, 32, 35]. Older women are often more aware of the importance of ANC due to previous pregnancies or exposure to health education programs over time [12, 16]. Additionally, they may have more stable socio-economic circumstances, facilitating access to and utilization of healthcare services [36]. Women with tertiary education generally have better access to health information, higher health literacy, and greater autonomy in decision-making regarding their healthcare, making them more inclined to seek ANC due to awareness of its benefits and potential risks associated with pregnancy [37].

Civil servants demonstrated higher odds of ANC utilization. This is in line with previous studies in Nigeria that found employment status and higher socioeconomic status to be associated with an increased likelihood of ANC utilization [7, 12, 27]. Other studies conducted in Africa have shown that ANC utilization is influenced by employment, health insurance coverage, and awareness of the benefits of using healthcare services [16, 35]. Prior studies reported that civil servants typically have stable employment, access to health insurance, and a regular income, thereby promoting the use of ANC services [16, 23].

Furthermore, women who are aware of the warning signs of pregnancy and have their husbands’ support during ANC are more likely to utilize ANC services. Studies have shown that pregnant women who recognize these warning signs are likely to be more aware of the potential risks associated with pregnancy and childbirth [38]. This awareness may motivate them to seek early intervention for any emerging issues or to obtain ANC as a preventive measure [39]. Support and involvement from a spouse or partner during ANC have been shown to positively influence a woman’s decision to seek medical care [40]. Husbands who accompany their spouses to ANC visits can enhance ANC utilization by providing emotional support, encouragement, and facilitating communication with healthcare providers [40].

This study is subject to some limitations. Given the complex nature of the subject matter and its association with various factors such as environmental, social, and cultural influences, it is possible that we may have overlooked some relevant questions. Secondly, the cross-sectional design presents challenges in establishing causation as it examines both cause and effect simultaneously. Thirdly, the study’s sample size is another limitation, as it is insufficient for the regression analysis given the number of predictors. A smaller sample size provides less information about the population, leading to greater variability in parameter estimates, overfitting, and wider confidence intervals. Furthermore, there is a potential for recall bias among respondents in certain instances. We recommend that future research should consider replicating this study in more diverse populations and different regions of Nigeria, particularly in areas characterized by lower utilization of ANC services and higher maternal and child mortality rates. This approach would facilitate the generation of robust evidence and the identification of factors responsible for appropriate ANC utilization.

### Conclusion

The current study investigated the factors influencing ANC utilization among women of childbearing age in Isari town, Jigawa State, employing a cross-sectional survey. The study revealed that a majority of participants possessed correct knowledge and exhibited a favorable attitude towards ANC services. However, slightly more than half of the participants availed themselves of full ANC services. Furthermore, determinants associated with ANC utilization included age, educational attainment, occupation, maternal awareness of pregnancy danger signs, and support from husbands for ANC. This research aims to equip policymakers with empirical evidence regarding the primary factors linked to ANC utilization, with the goal of enhancing attendance rates for ANC visits.

## Data Availability

All data are fully available from the corresponding author on reasonable request.
